# Laser-Assisted Diamond Turning for Anisotropy Suppression in Calcium Fluoride

**DOI:** 10.3390/mi17040425

**Published:** 2026-03-30

**Authors:** Enbo Xing, Jinsong Xue, Rongbiao Yang, Mingyue Wang, Huimin Zhou, Guohui Xing, Jianglong Li, Jiamin Rong, Huanfei Wen, Jun Tang, Jun Liu

**Affiliations:** 1 State Key Laboratory of Extreme Environment Optoelectronic Dynamic Measurement Technology and Instrument, North University of China, Taiyuan 030051, China; xiaoxing1228@126.com (E.X.); sz202306052@st.nuc.edu.cn (J.X.); yangrongbiao219@163.com (R.Y.); sz202306041@st.nuc.edu.cn (M.W.); zhmin0707@163.com (H.Z.); wenhuanfei@nuc.edu.cn (H.W.); 2 School of Semiconductors and Physics, North University of China, Taiyuan 030051, China; xinggh0913@163.com (G.X.); 15735657168@163.com (J.L.); juntang@nuc.edu.cn (J.T.)

**Keywords:** laser-assisted cutting, calcium fluoride (CaF_2_), anisotropy suppression, optical microcavities, brittle-ductile transition

## Abstract

This paper proposes the use of laser-assisted cutting technology to control the brittle–plastic transition of single-crystal CaF_2_ through local thermal softening, thereby suppressing its processing anisotropy. Nano-scratch experiments show that heating significantly increases the critical plastic cutting depth of each crystal plane and reduces the inter-plane differences. Based on this, laser-assisted ultra-precision turning was used to fabricate CaF_2_ optical microcavities with a surface roughness below 10 nm, achieving a maximum quality factor of ~7.79 × 10^7^, and significantly reducing the performance differences among different crystal orientations. The research indicates that this method can effectively promote uniform plastic flow on each crystal plane, providing an effective approach for the high-performance and consistent fabrication of anisotropic brittle optical components.

## 1. Introduction

Calcium fluoride (CaF_2_) crystals have become a key substrate material for high-power laser systems [1], ultraviolet lithography, and quantum optical devices due to their ultra-wide transmission window spanning from deep ultraviolet to mid-infrared wavelengths, low nonlinear refractive index, and excellent laser damage threshold. However, the significant anisotropy inherent in its face-centered cubic structure results in differentiated mechanical and thermal properties (such as elastic modulus, thermal expansion coefficient, and fracture toughness) across different crystal planes and orientations. This anisotropy poses a series of challenges in precision machining: For example, material removal rates on (111) and (100) crystal planes can differ by more than 30%, leading to non-uniform cutting forces and surface damage distribution. Furthermore, thermal expansion mismatches between different crystal orientations readily introduce residual stresses in devices, compromising their long-term service stability.

Laser-assisted processing technology offers a novel approach to regulating the machinability of hard and brittle materials [2]. Its core principle involves using a focused laser beam to locally preheat the workpiece, thereby reducing the material’s yield strength through thermal softening effects. This promotes plastic deformation and inhibits brittle fracture. In recent years, this technology has been successfully applied to the precision machining of materials such as silicon and sapphire [3]. However, there remains a significant research gap in applying laser-assisted cutting to CaF_2_ crystals and systematically investigating its mechanism for regulating crystal anisotropy. Existing studies predominantly focus on processing outcomes under single process parameters, lacking a systematic revelation of anisotropy evolution patterns under the multi-physics coupling effects of “temperature field-lattice orientation-cutting force.” More importantly, a clear correlation model has yet to be established for quantitatively mapping the improved surface anisotropy achieved through laser tuning to the core performance metrics of the final optical device, such as the quality factor Q of an optical resonator [4].

To address the aforementioned issues, this paper proposes a research framework integrating laser-assisted cutting with performance characterization. This approach aims to elucidate the mechanism by which the laser thermal field modulates the anisotropy of CaF_2_ crystal processing [5,6,7], thereby enabling the fabrication of high-performance optical microcavities. The main content of this study includes the following: (1) Systematically measuring the brittle–plastic transition depth on (100), (110), and (111) crystal planes at different temperatures (25–90 °C) and orientations through laser-assisted nano-scratch experiments [8], and quantitatively characterizing the evolution of anisotropy with temperature. (2) Optimize laser and cutting process parameters to achieve ultra-precision turning of CaF_2_ microcavities based on plastic-region removal. (3) Characterize the surface morphology and optical Q-factor of microcavities, establishing a correlation model linking “laser processing parameters → degree of anisotropic attenuation → enhancement of optical properties.” This provides theoretical foundations and process guidance for high-performance fabrication of anisotropic optical crystals.

## 2. Research on Scratch Tests

### 2.1. Critical Cutting Depth and Scratch Test Setup

**Figure 1:**
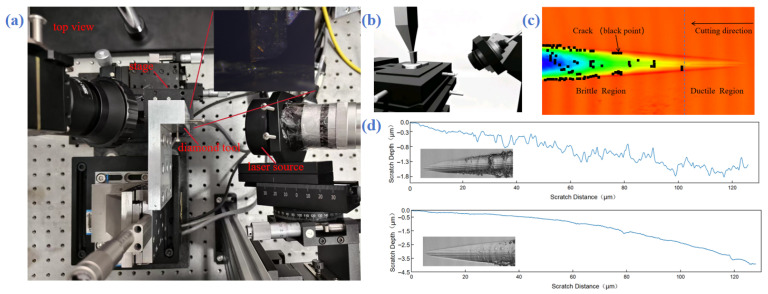
(**a**) Scratch apparatus comprising a movable angular displacement stage and high-precision displacement stages, with real-time monitoring provided by an infrared thermal imager and heating performed by a 1064 nm laser. (**b**) Schematic of laser-assisted scratch testing. (**c**) Confocal microscope scan of calcium fluoride surface after scratch testing (black dots indicate regions exhibiting brittle fracture). (**d**) Surface morphology comparison before and after laser-assisted scratching.

The ductile-to-brittle transition depth (DBT) is a critical process parameter and performance indicator in the field of ultra-precision machining, particularly in the processing of optical crystal materials [9,10]. In the fabrication of high-end components such as single-crystal calcium fluoride (CaF_2_) optical resonators, the precise understanding and control of DBT holds undeniable core significance. Its physical essence can be defined as: the critical depth threshold at which the material removal mechanism undergoes a fundamental transformation during nanoscale cutting of single-crystal materials [11,12,13]. Specifically, when the actual cutting depth is below this threshold, material removal is dominated by plastic flow; once it exceeds this threshold, the material removal process shifts to being dominated by brittle fracture. In the experiment, this represents the depth value at which the first brittle fracture occurs during the scratch process, with the scratch morphology observed and measured using confocal microscopy. Figure 1c displays the morphological features of a typical machined surface, where black markings indicate locations of brittle fracture or microcracks. Figure 1d further compares the scratch morphology on the (111) crystal plane under ambient temperature and laser-assisted heating conditions. At ambient temperatures, the machined surface exhibits numerous brittle fracture traces, whereas the surface heated by laser assistance appears smoother and more uniform overall. This indicates that elevated temperatures help suppress brittle fracture and promote plastic-range cutting.

As shown in Figure 1a, the scratch test setup employs a scratch apparatus composed of a movable angular displacement stage and a high-precision displacement stage [14]. To investigate the influence of temperature on the cutting process, a laser-assisted heating system was incorporated into the experiment. A continuous-wave laser from CNI with a wavelength of 1064 nm and a spot diameter of 3 mm was utilized to achieve rapid [15,16,17], controlled heating of localized areas on the sample surface. For real-time temperature monitoring, we employed an infrared thermal imager to provide immediate feedback. Figure 1b shows a schematic diagram of the scratch test details.

### 2.2. Research on Anisotropy

**Figure 2:**
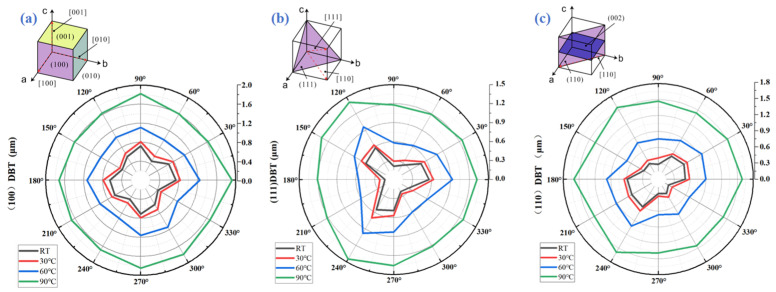
(**a**) (100) Depth of brittle–plastic transition at 25–90 °C in all directions of calcium fluoride crystals. (**b**) (111) Depth of brittle–plastic transition at 25–90 °C in all directions of calcium fluoride crystals. (**c**) (110) Depth of brittle–plastic transition at 25–90 °C in all directions of calcium fluoride crystals.

The inset at the top of Figure 2 shows a schematic diagram of the crystal structure of calcium fluoride (CaF_2_). Monocrystalline CaF_2_ exhibits a face-centered cubic structure. Its anisotropic atomic arrangement fundamentally determines the bonding strength and slip system availability across different crystal planes, as well as along distinct crystallographic orientations within the same plane. This crystallographic property directly influences the material’s local mechanical response, making the critical cutting depth (DBT) highly dependent on specific crystal plane orientations (such as (100), (110), and (111) planes) and the cutting direction relative to the crystal lattice orientation [5,6,7]. Therefore, understanding the crystal structure shown in Figure 2a provides the fundamental physics for in-depth analysis and prediction of the cutting behavior and anisotropic characteristics of single-crystal CaF_2_, as well as for optimizing cutting processes—including tool path planning and crystal orientation selection.

To systematically investigate the critical cutting depth of single-crystal calcium fluoride (CaF_2_) under varying crystal orientations and temperatures, this study employs nano-scratch testing combined with laser-assisted heating technology to explore its brittle–ductile transition behavior and anisotropic characteristics. The experimental system primarily consists of a high-precision displacement platform, a nano-scratch module, a real-time temperature measurement unit, and a laser heating device. The displacement system in the experiment includes a Sorebo manual displacement platform and a Newport high-precision dual-axis displacement stage [18,19], enabling precise positioning and feed of the sample within a two-dimensional plane. For temperature monitoring, an infrared thermal imager is employed to provide real-time monitoring and feedback of the sample surface temperature, ensuring the stability and reliability of temperature control. The experimental samples consisted of cylindrical single-crystal CaF_2_ crystals with a diameter of 20 mm and a thickness of 3 mm. Three representative crystal planes—(100), (110), and (111)—were selected for testing. The sample is secured to the displacement platform to ensure stable orientation during the scratch test.

On each test crystal surface, rotational cutting was performed at an angle of 30° relative to the crystal orientation to comprehensively evaluate the impact of different crystal orientations on cutting performance [18,19,20,21,22,23]. The scratch test employed a single-crystal diamond tool with a tip radius of 0.02 mm, a rake angle of 0°, a scratch slope of 2°, and a constant feed rate of 0.06 mm/min.

The anisotropic polar plots shown below Figure 2 summarize the distribution of critical cutting depths for each crystal plane at different temperatures and under various scratching directions [10,24,25,26,27,28]. The results reveal that the polar coordinate curves of critical cutting depths for the (100) crystal plane exhibit polygonal shapes, with significant numerical differences between crystal orientations. This indicates pronounced anisotropy in the mechanical response of this crystal plane under varying cutting directions (111). The anisotropic characteristics of the crystal surface are particularly pronounced, with critical cutting depth exhibiting significant variation within specific angular ranges. This is primarily attributed to the (111) plane being a close-packed atomic plane, where the bond strengths and cutting resistances of atoms on different crystal orientations exhibit significant differences (110). The critical cutting depth distribution on the crystal plane also exhibits non-uniformity, reflecting the coupling effect between the atomic arrangement structure of this crystal plane and the cutting direction.

As the temperature rises from room temperature to 90 °C, the differences in critical cutting depth curves across crystal planes gradually diminish, and the degree of anisotropy significantly decreases. Specifically, at higher temperatures (such as 90 °C), the polar coordinate curves of each crystal plane tend to become “smoother,” and the critical cutting depth difference between different crystal orientations is significantly reduced. The underlying mechanism can be explained as follows: increased temperature intensifies the thermal motion of atoms within the crystal, weakening the directional constraints imposed by crystal orientation on interatomic bonding forces. This alleviates the mechanical property variations caused by crystal orientation, resulting in a reduction of anisotropic characteristics.

In summary, this study employed nano-scratch experiments and laser-assisted heating technology to reveal the anisotropic behavior and temperature dependence of the critical cutting depth in single-crystal CaF_2_ across different crystal planes and temperatures. These findings provide experimental evidence for understanding its brittle–plastic transition mechanism and enabling efficient precision machining.

## 3. Laser-Assisted Precision Turning of Calcium Fluoride

### 3.1. Laser-Assisted Single-Point Diamond Cutting

Although we obtained information on the critical cutting depth for specific directions in orthogonal cutting experiments, the cutting direction continuously changes during cylindrical turning of high-precision calcium fluoride crystal resonators. Therefore, in addition to controlling the cutting depth within the ductile cutting range, it is also necessary to experimentally determine the optimal turning parameters to achieve high Q-value micro-resonators. We prepared calcium fluoride crystal resonators with end-face orientations of (100), (110), and (111).

**Figure 3:**
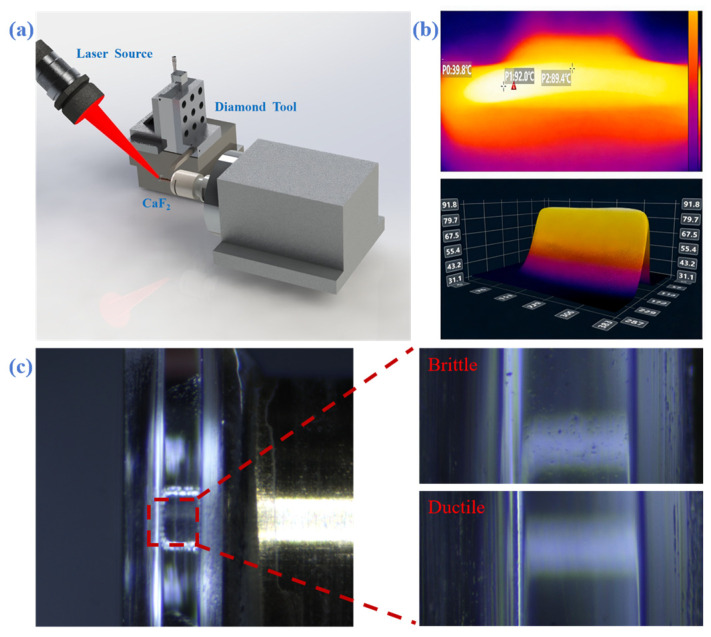
(**a**) Laser-assisted cutting system comprising a highly stable air-bearing spindle, a 1064 nm laser, and a Newport high-precision dual-axis displacement stage. (**b**) Two-dimensional and three-dimensional thermal images of a calcium fluoride crystal cavity under laser heating (The brighter the color, the higher the temperature). (**c**) Surface condition of calcium fluoride after cutting (inset shows surface features of brittle and ductile regions formed post-cutting).

During the process, we employed a stage equipped with Hualing Superhard’s diamond cutting tools to machine the calcium fluoride resonator cavity. As shown in Figure 3a, we secured a 0.5 × 1.0 mm calcium fluoride crystal cavity onto a 3 mm diameter brass pillar using UV adhesive. For cavity mounting, we employ an air-bearing spindle with a vibration error of ±50 nm to support high-precision machining. By adjusting the angle and distance via the displacement stage, we control the laser spot size to 3 mm, preventing crystal cavity fragmentation caused by excessive laser density. Infrared thermal imaging revealed the cavity temperature reached 90 °C before machining commenced, as shown in Figure 3b. Our cutting process was divided into three stages. Initial rough turning was performed to establish the required radius. It should be noted that this initial rough turning is performed in the brittle zone. Then perform pre-finishing to remove large cracks that appear in brittle mode, with a thickness reduction of 8 µm. Finally, ductile mode precision cutting achieved ultra-precision turning under the following conditions: 500 min^−1^ rotational speed, 0.1 mm/min feed rate, 50 nm cutting depth, and 2 µm removal thickness [29]. We can see that cracks exceeding 10 µm in depth that occur during rough turning cannot be eliminated through pre-finishing or finish cutting. Although the greater removal thickness results in lower production efficiency, it allows for the removal of deep unwanted cracks.

Based on the results obtained from the previous scratch test, we set the precision cutting depth to 100 nm. To achieve a smooth surface, other process factors such as feed rate, tool radius, and rotational speed should be considered. In particular, previous research reports indicate that feed rate significantly impacts the quality of machined surfaces, as does the combination of tool radius and cutting depth. These studies indicate that rapid feed rates result in brittle-mode cutting when the cutting depth remains below the critical threshold. Therefore, when producing smooth surfaces, we selected slower feed rates (≤1 mm/min).

For roughing and finishing operations, we employed single-crystal diamond tools with tool tip radii of 0.05 mm and 0.1 mm, featuring a 0° front angle and a 10° clearance angle. In tool selection, a smaller cutting edge radius reduces the contact area between the tool and material during external turning, which helps minimize excessive cutting forces and thereby improves surface finish. However, tools with smaller cutting edge radii are more fragile and have shorter tool life; therefore, in this work, we employed different tools for the rough turning and finish turning stages. Observation of the machined surface under an optical microscope is shown in Figure 3c. In the microscopic image, the difference between the rough turning area and the finish turning area is very distinct.

### 3.2. Surface Comparison After Precision Machining

To systematically evaluate the optimization effect of laser-assisted cutting on the surface quality of single-crystal calcium fluoride (CaF_2_), particularly its role in regulating crystal anisotropy, this study performed multi-scale, multi-dimensional morphological characterization of the processed surfaces. Our characterization process follows a macro-to-micro, qualitative-to-quantitative approach: First, all scratched areas undergo preliminary rapid scanning and observation using an optical microscope (Olympus BX53M) to identify representative regions and conduct a qualitative assessment of surface macro-integrity (e.g., presence of long cracks, chipped edges, etc.) [30]. Subsequently, based on preliminary localization using optical microscopy, atomic force microscopy (AFM, Bruker Dimension Icon, Bruker, Billerica, MA, USA) was employed to perform high-resolution three-dimensional topography scans of selected regions in tapping mode, typically covering an area of 20 × 20 μm^2^.

**Figure 4:**
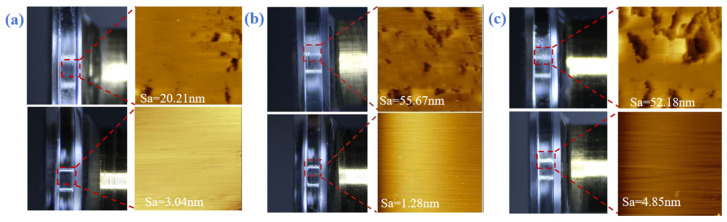
(**a**) Comparison of surface roughness on the (100) crystal plane with and without laser assistance. (**b**) Comparison of surface roughness on the (111) crystal plane with and without laser assistance. (**c**) Comparison of surface roughness on the (110) crystal plane with and without laser assistance.

By comparing the AFM topography images and surface roughness data (represented by arithmetic mean height Sa) of various crystal planes after conventional cutting and laser-assisted heating cutting [31], the significant improvement in surface quality achieved by laser-assisted cutting and its weakening effect on anisotropy are clearly demonstrated, as shown in Figure 4a–c.

(100) Crystal plane: AFM topography and roughness data confirm the anisotropic nature of this crystal plane under nano-cutting conditions. At room temperature during cutting, the surface morphology exhibits distinguishable differences: scattered pits caused by brittle microfractures are visible in some directions, while relatively continuous plastic grooves appear in others. The corresponding Sa values, after laser-assisted heating (90 °C), exhibited a significant enhancement in plasticity due to the thermal softening effect. This resulted in cutting along different crystal orientations tending toward a fully plastic deformation mode. As a result, not only did the Sa values across all directions show a significant overall decrease compared to room temperature, but the differences in Sa values between different directions also converged substantially. The roughness polar curve transitions from a slightly quadruple-symmetric “quasi-square” distribution at room temperature to a more circular shape at elevated temperatures, visually demonstrating that laser heating effectively homogenizes the machining response across different orientations of this crystal plane.

(111) Crystal plane: As the crystal plane exhibiting the most pronounced anisotropy, its surface morphology is highly sensitive to crystal orientation during conventional machining. Within certain specific angular ranges, the surface is dominated by dense brittle fractures, revealing numerous deep valleys and steep peaks under optical microscopy. Conversely, along other “soft” orientations, relatively continuous plastic grooves can be observed. Laser-assisted cutting has revolutionized this situation. At 90 °C, surfaces in all cutting directions exhibit a continuous and flat morphology dominated by plastic deformation. Although minor undulations persist, the sharp discontinuities are no longer present. Roughness data indicate that, not only have Sa values across all directions generally decreased below 10 nm, but the disparity between maximum and minimum values has also narrowed significantly. This demonstrates the effective suppression of the laser thermal effect on the strong directional dependence of the densely packed surface.

(110) Crystal Face: At room temperature, this crystal face exhibits moderate anisotropy. Although the variation in roughness with angle is less pronounced than that of the (111) crystal face, it still displays a certain degree of non-uniform distribution. Following laser-assisted heating, the improvement in surface quality is equally remarkable. AFM images reveal that the previously alternating discontinuous brittle fracture zones in different directions have been replaced by more coherent, directional micro-nano-scale plastic flow lines. These flow lines may be associated with specific slip systems activated at elevated temperatures. The overall level of roughness Sa and its anisotropic variation decreased simultaneously, further confirming that elevated temperatures promote more uniform plastic material removal behavior across crystal orientations.

### 3.3. Optical Properties of Microcavities After Cleaning

**Figure 5:**
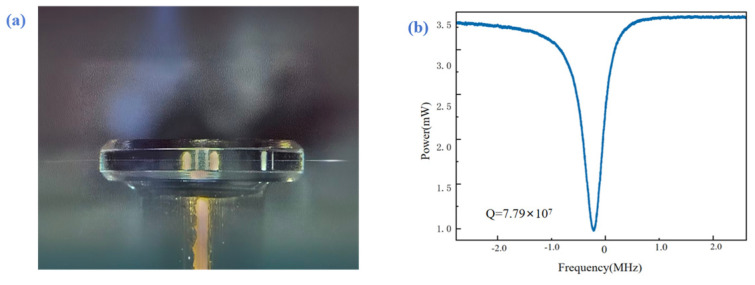
(**a**) Fiber Optic coupling testing; (**b**) Q-factor of an ultra-precision-turned crystal micro-resonator.

To evaluate the final optical performance, we measured the “laser-assisted cutting + simple cleaning” process. As shown in Figure 5, optical characterization results show that after cleaning, the Q-factor of the (100) crystal microcavity can reach approximately 7.79 × 10^7^ [29].

This excellent optical performance stems from laser-assisted cutting, which suppresses anisotropic brittle fracture at its source, forming a uniform plastic-worked surface with consistent defect depth. Conventional polishing exhibits low repair efficiency on surfaces with pronounced anisotropy and readily introduces damage. Therefore, experiments demonstrate that laser-assisted cutting fundamentally achieves ultra-high and consistent Q-values in microcavities by thermally softening and weakening anisotropy.

## 4. Discussion

This study systematically investigates the anisotropic control of laser-assisted cutting technology on single-crystal calcium fluoride crystal processing and its mechanism for enhancing microcavity optical properties. Through nano-scratch experiments, the critical cutting depth evolution patterns for the three primary crystal planes—(100), (110), and (111)—were quantitatively obtained across different temperatures (from room temperature to 90 °C) and crystal orientations. Experimental results demonstrate that the laser-induced localized thermal softening effect significantly promotes plastic deformation removal in materials, universally enhances the critical cutting depth across all crystal planes, and effectively mitigates their inherent machining anisotropy. Among these, the (111) crystal plane—which exhibits the most pronounced anisotropy—shows the most significant improvement.

This study achieved ultra-precision plastic-range turning of CaF_2_ crystal microcavities. The laser-assisted strategy not only suppressed brittle fracture at its source, producing an ultra-smooth surface with a surface roughness below 10 nm, but also established an isotropic ideal substrate for subsequent finishing processes. Subsequent moderate mechanical polishing on this homogenized surface can further enhance the microcavity quality factor to approximately 7.79 × 10^7^, demonstrating the effectiveness of laser-assisted processing.

In summary, this study elucidates an effective method for regulating crystal anisotropy through active thermo-mechanical coupling field control, progressing from mechanism exploration to process validation. This strategy not only provides a systematic theoretical basis and key technological support for overcoming the challenges in fabricating high-performance devices from brittle optical crystals such as CaF_2_, but also offers a novel approach for the precision processing of other anisotropic functional materials. Future work may further explore new mechanisms, such as ultrafast laser-assisted techniques, to achieve more precise real-time control over material removal and surface formation processes.

## Data Availability

The experimental data that support the findings of this study, including nano-scratch test results, surface roughness measurements, and quality factor (Q-factor) values, are available from the corresponding author upon reasonable request.
